# Episodic ataxias in children and adolescents: Clinical findings and suggested diagnostic criteria

**DOI:** 10.3389/fneur.2022.1016856

**Published:** 2022-10-24

**Authors:** Filipp Maximilian Filippopulos, Lutz Schnabel, Konstanze Dunker, Ralf Strobl, Doreen Huppert

**Affiliations:** ^1^German Center for Vertigo and Balance Disorders (DSGZ), University Hospital, Ludwig-Maximilians-Universität, Munich, Germany; ^2^Department of Neurology, University Hospital, Ludwig-Maximilians-Universität, Munich, Germany

**Keywords:** episodic ataxia, spinocerebellar ataxia 27, ocular motor disturbances, children, adolescents, vertigo, dizziness

## Abstract

**Background:**

The main clinical presentation of episodic ataxias (EAs) consists of vertigo and dizziness attacks lasting for minutes to hours with widely varying accompanying symptoms. The differentiation of EA and episodic vertigo/dizziness syndromes in childhood and adolescence such as vestibular migraine (VM) and recurrent vertigo of childhood (RVC) can be challenging. Furthermore, only few prospective studies of children/adolescents with EA are available.

**Objective:**

This study aims to characterize clinical and instrument-based findings in EA patients under 18 years of age, to delineate the clinical and therapeutic course in EA, and to present potentially new genetic mutations. Furthermore, the study aims to differentiate distinct characteristics between EA, VM, and RVC patients.

**Methods:**

We prospectively collected clinical and instrument-based data of patients younger than 18 years, who presented at the German Center for Vertigo and Balance Disorders (DSGZ) at the LMU University Hospital in Munich with EA, VM, or RVC between January 2016 and December 2021. All patients underwent a comprehensive evaluation of neurological, ocular-motor, vestibular and cochlear function, including video-oculography with caloric testing, video head impulse test, vestibular evoked myogenic potentials, posturography, and gait analysis.

**Results:**

Ten patients with EA, 15 with VM, and 15 with RVC were included. In EA the main symptoms were vertigo/dizziness attacks lasting between 5 min and 12 h. Common accompanying symptoms included walking difficulties, paleness, and speech difficulties. Six EA patients had a previously unknown gene mutation. In the interictal interval all EA patients showed distinct ocular-motor deficits. Significant differences between EA, VM, and RVC were found for accompanying symptoms such as speech disturbances and paleness, and for the trigger factor “physical activity”. Furthermore, in the interictal interval significant group differences were observed for different pathological nystagmus types, a saccadic smooth pursuit, and disturbed fixation suppression.

**Conclusion:**

By combining clinical and ocular-motor characteristics we propose diagnostic criteria that can help to diagnose EA among children/adolescents and identify patients with EA even without distinct genetic findings. Nevertheless, broad genetic testing (e.g., next generation sequencing) in patients fulfilling the diagnostic criteria should be conducted to identify even rare or unknown genetic mutations for EA.

## Introduction

The diagnoses behind recurrent vertigo attacks in children and adolescents are manifold and often pose a major diagnostic challenge even for experienced clinicians. The most common diagnoses include vestibular migraine (VM) and the associated disorder of “Recurrent Vertigo of Childhood” (RVC), whereas central causes such as episodic ataxia (EA) are less frequent (1–3). The core features of these diseases may be very much alike and include recurrent attacks of vertigo (sensation of spinning of the environment)/dizziness (various sensations of body orientation and position), headaches, and different trigger-factors, as well as an inconclusive clinical examination, at least in early stages. Furthermore, overlap syndromes between e.g., migraine and EA (4, 5), epilepsy and EA (6), or progressive and episodic ataxias (e.g., spinocerebellar ataxia type 27 and EA 9) (7) have been described. Especially diseases with a rare occurrence such as EA are sometimes difficult to diagnose. Nevertheless, due to the direct therapeutic relevance (e.g., acetazolamide or 4-aminopyridine for EA, magnesium for VM), different prognosis (often progressive in EA, benign in RCV and VM), and impact on family planning (EA mostly has an autosomal dominant inheritance pattern), it is important not to miss such differential diagnoses of episodic vertigo syndromes.

Episodic ataxias are hereditary chanellopathies with symptoms mainly attributable to a cerebellar dysfunction, that are genetically and phenotypically heterogeneous (8), which further complicates the diagnostic approach. To date, nine phenotypes and genotypes of EA have been described (EA 1–9) (7, 9), with EA 2 being most common (10). EA 1 is typically caused by mutations of the potassium channel Kv1.1-encoding gene KCNA1 on chromosome 12q13 (11), and EA 2 by mutations in the CACNA1A gene on chromosome 19p13 which encodes the Cav2.1 subunit of the P/Q-type voltage-gated calcium channel (5). Although EAs usually have an autosomal-dominant inheritance pattern (7–9), spontaneous mutations have been described (4, 12), so that affected children/adolescents might not always have a positive family history, again complicating the correct diagnosis. The age of onset, disease characteristics, and symptom constellation during and between attacks is highly variable even in patients with the same gene mutation causing the EA (6, 10). No causative treatments are available for any EA syndrome, but there are some symptomatic treatment options available for EA 1 and 2 such as acetazolamide and 4-aminopyridine (10), that may have a significant impact on the patients' quality of life.

Since the knowledge of the clinical spectrum as well as typical instrument-based findings in patients with EA are the key for early diagnosis and specific treatment, we prospectively examined ten children/adolescents with episodic ataxia syndromes. We describe clinical and instrument-based findings, treatment response, and clinical course. Furthermore, to depict the most important features for diagnosing EA, we compared clinical and instrument-based findings between EA patients and patients with the most common episodic vertigo syndromes in children/adolescents, namely VM and RVC.

## Methods

### Subjects and clinical/instrument-based evaluation

All children/adolescents younger than 18 years of age who presented at the German Center for Vertigo and Balance Disorders (DSGZ) at the LMU University Hospital in Munich between January 2016 and December 2021 were screened for inclusion. All patients with an episodic ataxia syndrome were included in the study. Furthermore, patients younger than 18 years with the final diagnosis of vestibular migraine (VM) or RVC according to the diagnostic criteria of the Bárány Society (13) were included in the study as comparative groups. All patients who were recruited before the publication of the diagnostic criteria in 2021 were reevaluated and only included for further analysis, if the diagnostic criteria for VM or RVC were fulfilled. Written informed consent was obtained from all participants included in the study.

Due to the lack of evidence on distinct clinical and instrument-based findings in patients with EA, an extensive, standardized work-up was designed to broadly evaluate clinical features and instrument-based findings these patients. The work-up was comprised to include most of the available neuro-otological examinations for the assessment of the peripheral and central vestibular system. Following examinations were performed:

A) Medical history (including duration and quality of symptoms, accompanying symptoms, trigger factors, family history, medication).
B) Clinical examination of the vestibular function including neuro-otological and neuro-ophthalmological examination (including head impulse test, test for spontaneous nystagmus, provocation nystagmus, positional nystagmus, skew deviation, smooth pursuit, saccades, gaze-holding, fixation suppression of the vestibulo-ocular reflex, hearing).
C) Neurological status (including motor, sensory, coordination, cranial nerve, cognitive function assessment).
D) Instrument-based assessment:
  a. Video-oculography with caloric irrigation was conducted with cold (30°C) and worm (44°C) water on both sides to evaluate the low frequency function of the vestibular system
  b. Video-Head-Impulse-Test (vHIT) was performed on a standard commercial v-HIT system to evaluate the high frequency function of the vestibular system
  c. Ocular and cervical Vestibular-Evoked Myogenic Potentials (c/o VEMP's) were analyzed for the evaluation of utricular and saccular function respectively
  d. Auditory-Evoked Potentials (AEP's) for the evaluation of the hearing function
  e. Posturography and gait analysis was performed to measure body sway and gait patterns
  f. Testing of subjective visual vertical, and fundus photography.
D) Clinical follow-up for patients with EA syndrome.

### Statistical analysis

We report mean and standard deviation for continuous variables, and absolute frequency and the relative frequency as percentages for categorical variables. Group comparison is based on the chi-squared test for categorical data and on the likelihood ratio test for continuous variables.

Two-tailed *p*-values <5% were considered as statistically significant. As this is an exploratory study, no correction for multiple testing was done. R 4.1.2 was used for statistical analyses (14).

## Results

**Figure 1:**
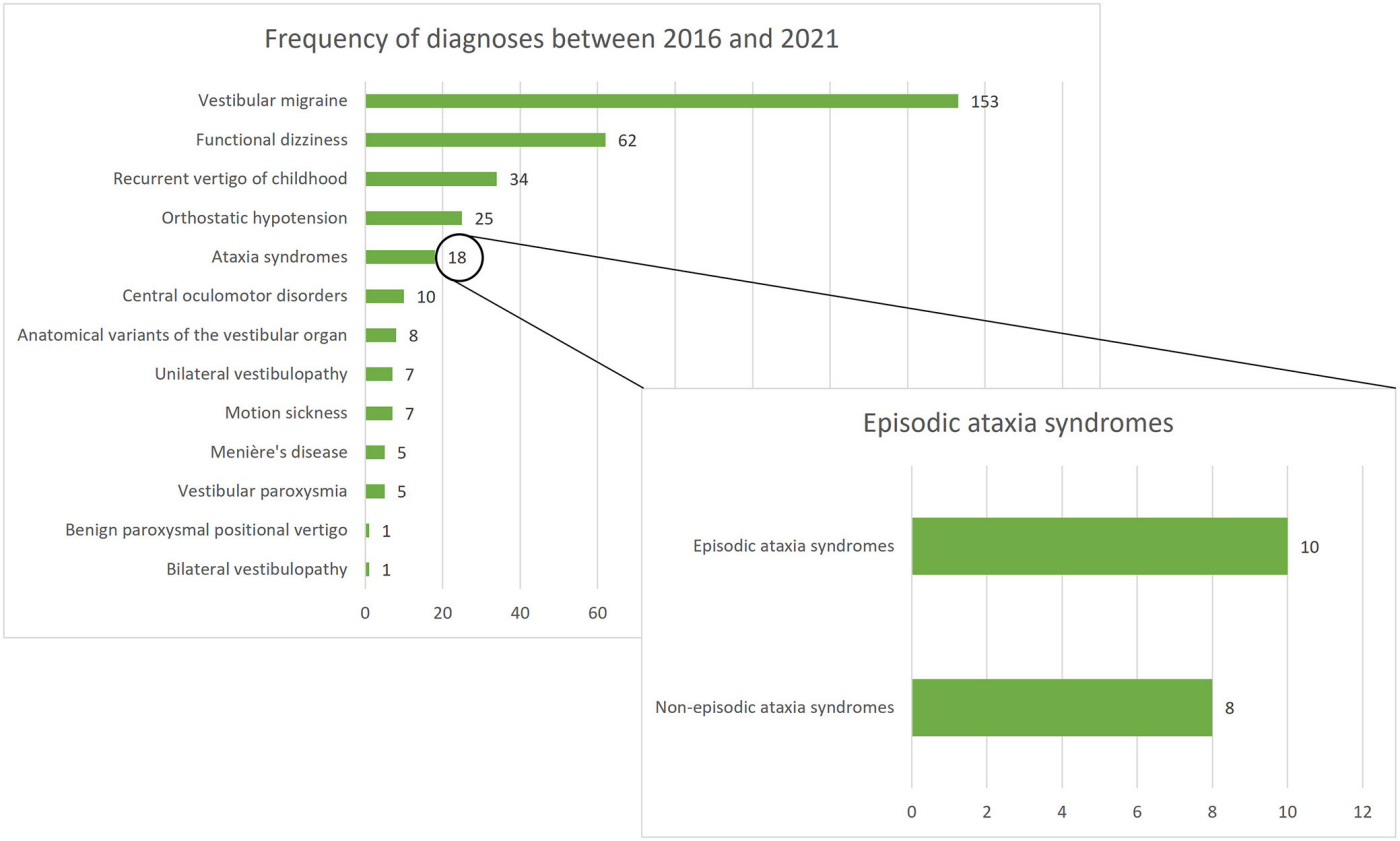
Frequency of diagnoses among 336 children/adolescents presenting at the German Center for Vertigo and Balance Disorders (DSGZ) between 2016 and 2021. Ataxia syndromes are the fifth most frequent diagnosis with episodic ataxias being the leading subgroup.

Between January 2016 and December 2021, 336 patients under the age of 18 were screened for inclusion. Of all screened children/adolescents, ataxia syndromes represented the fifth most common diagnosis following VM, functional dizziness, RVC, and orthostatic hypotension. A total of 18 children/adolescents with ataxia syndromes presented in the period mentioned, 10 of whom had an episodic ataxia syndrome and were included in the present study (see Figure 1). Furthermore, 15 age- and gender- matched children and adolescents with VM and RVC were included in the comparative groups.

### EA characteristics

Of the 10 children/adolescents with EA, seven had a genetically confirmed EA 2, one had a genetically confirmed EA 1, one a genetically confirmed EA 9, and one child had a suspected EA 1. In the latter child, a mutation in the CACNA1A gene was ruled out; further testing for mutations e.g., in the KCNA1 gene was not possible due to insurance restrictions (Table 1). Nevertheless, the child was included in the EA group due to the distinct clinical and instrument-based findings (see Tables 2, 3), as well as due to the fact, that in a considerable amount of children and adolescents with EA, no mutation at all is found (8). Of the 10 children/adolescents five had a spontaneous mutation defined as no genetic marker in tested family members nor a positive family history (including the suspected EA 1 child). Six children/adolescents (five with EA 2, one with EA 1) had a new mutation, that has not been described in common gene databases such as the University of California Santa Cruz (UCSC) genome browser, the human gene mutation database (HGMD), ClinVar at the National Center for Biotechnology Information (NCBI), and global variome shared Leiden Open Variation Database (LOVD) (see Table 1).

**Table 1:** Genetic and clinical course characteristics of 10 children/adolescents with episodic ataxia.

**Nr**.	**EA**	**Symptom onset**	**Genetic mutation**	**Previously unknown mutation**	**Spontaneous mutation**	**Developmental delay**	**SARA score (attack free interval)**	**Attack frequency/month before treatment**	**Treatment (in order of prescription)**	**Attack frequency/month after treatment**	**Other treatment response**
1	1	5 yrs	c.555C>g in the KCNA1 gene	Yes	Yes	No	0	4	Acetazolamide, Magnesium	4	None
2	(1)	4 yrs 7 mos	Yet Unknown, no mutation in the CACNA1A gene		Probably (no family history)	No	0	30	None	-	-
3	9	11 yrs 8 mos.	Microdeletion 13q33 in the first Exon of the FGF14 gene	No	Yes	Yes	8	30	Magnesium, behavioral recommendations	6	reduced SARA score (4)
4	2	8 yrs	c.5035C>T; p.(Arg1679Cys) in the Exon 32 of the CACNA1A gene	No	Yes	No	0	2	4-Aminopyridine	1	None
5	2	0 yrs 9 mos	c.4300C>T; p.Arg1434Trp in the CACNA1A gene	Yes	No	Yes	0	1	Acetazolamide	1	None
6	2	12 yrs	c.1949delA; p.Asn650Thrfs*9 in the CACNA1A gene	Yes	Yes	No	0	1	None	-	-
7	2	12 yrs 6 mos	c.5570G>A (p.Arg1857Gln) in the CACNA1A gene	Yes	No	No	0	5	None	-	-
8	2	10 yrs	c.3370G>A; p.(Ala1124Thr) **AND** c.1011delG; p.(Trp337Cysfs*16), both in the CACNA1A gene	Yes / Yes	Yes	Yes	3	8	Acetazolamide, 4-Aminopyridine	4	Reduced SARA score (1)
9	2	1 yr 6 mos	c.4095_4096delGT (Stop-Kodon) in the CACNA1A gene	Yes	No	Yes	1,5	4	Magnesium, Acetazolamide, 4-Aminopyridine		None
10	2	8 yrs	c.5419-1G>A in the CACNA1A-Gen	No	No	No	0	8	Acetazolamide	1	attack duration ↓ (60 → 5 min)

Six children/adolescents showed a novel disease-causing mutation. Five children/adolescents had a spontaneous mutation. Not all patients responded to treatment with acetazolamide or 4-Aminopyridine.

EA, Episodic ataxia type; SARA, Scale for the Assessment and Rating of Ataxia.

**Table 2:** Detailed clinical characteristics of the vertigo/dizziness attacks of children and adolescents with episodic ataxia.

**Nr**.	**EA**	**Main symptom**	**Attack freq**.	**Attack dur**.	**Accompanying symptoms**									**Trigger**	
		***Vert. (V) Dizz. (D)***	***per month***	***in min*.**	**Head-ache**	**Speech disturb**	**Limb ataxia**	**Walking difficulties**	**Photo-/Phono-phobia**	**Double vision**	**Oscill- opsia**	**Nausea/Vomiting**	**Paleness**	**Sport**	**Stress**
1	1	D	4	20	n	y	n	y	n	n	y	y	n	y	y
2	(1)	V	30	10	n	n	n	y	n	n	y	y	y	y	n
3	9	V	30	30	y	n	n	n	y	y	n	y	y	y	n
4	2	V	2	120	n	n	n	y	n	n	n	y	y	y	y
5	2	V	1	5	n	n	n	y	n	y	y	n	n	y	n
6	2	D	1	30	y	n	n	y	y	n	n	y	n	n	n
7	2	V/D	5	40	n	y	n	y	n	n	n	y	n	y	n
8	2	V	8	720	y	y	n	y	n	n	y	y	y	y	y
9	2	D	4	150	n	n	y	n	n	n	n	y	y	n	n
10	2	D	8	60	n	n	y	y	n	n	n	n	n	y	n

Attack frequency varied between 1 to 30 times per month, attack duration from 5 to 720 min. The most frequent accompanying symptom “walking difficulties” was often described as “having to lie down” or “not being able to stand up”. Physical activity (“sport”) was the most common trigger factor.

D, Dizziness; V, Vertigo; n, no, y, yes.

**Table 3:** Ocular-motor and instrument-based findings in the attack-free (interictal) interval in children and adolescents with episodic ataxia.

**Ocular-motor findings**										
**Nr**.	**EA**	**Strabismus**	**Nystagmus**				**Saccadic smooth pursuit**		**Optokinetic nystagmus**	**Fixation suppression**
		***-phoria***	**Provocation**	**Gaze holding**	**Down-/Up-beat**	**Rebound**	**Horizontal**	**Vertical**		
1	1	Exo-	n	n	n	n	+++	+++	norm	norm
2	(1)	Eso-	n	y	n	n	+++	+++	norm	path
3	9	Eso-	y	y	D	y	+++	+++	path	path
4	2	Eso-	y	y	D	y	+++	+	path	path
5	2	Exo-	y	n	D	y	+++	+++	path	path
6	2	Exo-	y	y	U	n	+	+	path	norm
7	2	n	n	n	n	n	+	+	norm	norm
8	2	Eso-/Exo-	n	y	D	n	+	+++	path	path
9	2	n	n	n	U	n	+++	++	path	norm
10	2	n	n	n	n	n	norm	+	norm	norm
**Instrument-based findings**										
**Nr**.	**EA**	**Gait analysis**		**Caloric irrigation**		**VEMP's**	**AEP**	**Audio**	**Posturography**	**MRI**
1	1	-		-		norm	-	-	norm	-
2	(1)	-		norm		norm	norm	norm	functional	norm
3	9	atactic		-		-	-	-	norm	norm
4	2	functional		-		-	-	-	norm	norm
5	2	atactic		norm		norm	norm	norm	functional	norm
6	2	-		norm		norm	-	norm	functional	norm
7	2	-		norm		norm	norm	norm	-	-
8	2	-		norm		-	norm	-	functional	norm
9	2	-		-		-	-	-	functional	-
10	2	-		-		norm	-	-	norm	norm

The most frequent findings were the presence of a nystagmus and a saccadic smooth pursuit. Except atactic and functional patterns in the gait analysis and posturography, no distinct pathological findings were recorded in the instrument-based examinations.

VEMPS, vestibular evoked myogenic potentials; AEP, acoustic evoked potentials; Audio, audiogram; MRI: magnetic resonance imaging; n, no; y, yes; U, up-beat; D, down-beat; +, gain 0.65–0.85; ++= gain 0.45–0.65; +++, gain < 0.45; norm, normal; path, pathological.

The mean age at symptom presentation in children/adolescents with EA was 7.4 ± 4.3 years. The earliest symptom manifestation was in the ninth month in one child with EA 2 and the latest at the age of 12 years and 6 months, also in an adolescent with EA 2.

Attack frequency varied between daily attacks (in EA 9 and suspected EA 1) and attacks once a month (mean ± sd = 9.3 ± 11.2). The Scale for the Assessment and Rating of Ataxia (SARA) in the attack-free interval was increased in three children/adolescents (for details see Table 1).

In a vertigo/dizziness attack, the most common accompanying symptoms were “nausea/vomiting” (80%) and experiencing “walking difficulties” (80%), which were often described as the “inability to walk” and “having to lie down” until the attack is over. Furthermore, “paleness” (50%) and “oscillopsia” (40%) were commonly described. The most frequent trigger for an attack was physical activity (“sport”, 80%), followed by psychosocial stress (30%), while two children/adolescents (20%) did not report any trigger factors. Detailed findings are listed in Tables 2, 3.

The broad ocular-motor evaluation showed in all patients with EA an impaired smooth pursuit, in most cases with a medium to highly reduced smooth pursuit gain at 0.1 and 0.2 Hz (Hertz). Furthermore, 70% of the patients had a pathological finding in the nystagmus examination (see Table 3). The optokinetic nystagmus was impaired in 60% of patients and fixation suppression in 50%. Overall, 70% of patients had more than one ocular-motor abnormality. No pathological findings were detected in additional instrument-based examinations such as caloric irrigation, VEMPS, AEPs, and audiogram.

#### EA disease course

The mean follow up of the patients with EA was 2.7 years. Seven children/adolescents received treatment with at least one medication, two declined treatment and one with genetically not confirmed EA 1 was not offered any specific medication. Medical treatment was administered for at least 6 weeks and then changed or discontinued if no therapeutic effect was noted. Initial treatment was chosen according to the side-effect profile of each substance and according to patient/parents' wishes; dosage was adapted to weight for acetazolamide (8–30 mg/kg), 4-aminopyridine was restricted to a maximum of 2 doses of 10 mg per day. In four cases acetazolamide was prescribed but led to a reduction of the frequency or duration of the vertigo attacks in only one case. 4-Aminopyridine was administered in three children/adolescents (the youngest being 1 year and 6 months old) and led to a decrease in attack frequency and duration in two cases. Magnesium which was administered in three patients did not influence vertigo attacks. In the adolescent with EA 9, especially the behavioral recommendations (in combination with magnesium) led to a significant decrease in attack frequency and severity, but also to a reduced SARA score (see Table 1).

### Comparison of EA with VM and RVC

Table 4 shows the results of the comparison between EA, VM, and RCV in detail. A positive family history of genetically determined EA and delayed motor and/or mental development occurred significantly more often (*p* = 0.0012) in EA patients compared to patients with VM and RVC (40 vs. 0%). The attack characteristics (type of vertigo, attack frequency, attack duration) did not differ between the three diagnoses. Accompanying symptoms such as “speech disturbances” and “paleness” differed significantly (*p* = 0.0429 and *p* = 0.0212 respectively) between groups, with the highest frequency observed in EA patients (30 and 50% respectively), while “headache” (*p* <0.0001) and “photo-/phonophobia” (*p* < 0.0001) were most often observed in children/adolescents with VM (80% each). Furthermore, physical activity (“sport”) as a trigger factor occurred more often in EA patients (60%, *p* = < 0.0001). Provocation, gaze-holding, and down-beat nystagmus were more frequently observed in EA, but also occurred, in a milder form, in some patients with VM, but no patients with RVC. Similarly, an impaired smooth pursuit was observed in all EA patients, in 47% of VM patients, and in 7% of RVC patients, significantly differing between the groups (*p* < 0.0001). In all VM and RVC patients with an impaired smooth pursuit, the gain was only slightly reduced (gain between 0.65 and 0.85) in contrary to the EA patients (see above).

**Table 4:** Comparison of clinical and instrument-based findings between children and adolescents with episodic ataxia, vestibular migraine, and recurrent vertigo of childhood.

	**Episodic Ataxia (*n* = 10)**	**Vestibular Migraine (*n =* 15)**	**Recurrent vertigo of childhood (*n =* 15)**	***p*-value**
Age at diagnosis [years; (min.; max.)]	11 (3.75; 15.8)	11.5 (5.5; 17)	7.0 (2,75; 10,6)	**<0.0001**
Years to diagnosis	3.7 ± 2.74 (0.3; 7.8)	1. ± 1.8 (0.3; 5.8)	1.2 ±0.8 (0.2; 3.6)	0.0017
Gender (f:m)	7:3	10:5	9:6	0.8637
Delayed motoric/cognitive development	4 (40%)	0 (0%)	0 (0%)	**0.0098**
Family history for EA	4 (40%)	0 (0%)	0 (0%)	0.0013
Family history for migraine	2 (20%)	10 (67%)	7 (47%)	0.0726
**Attack characteristics**				
Form of vertigo (dizziness: vertigo)	5:5	4:11	3:12	0.2271
Mean attack frequency [days/month; (min.; max.)]	9.3 ± 11.2 (1; 30)	19 ± 17.8 (1; 60)	12.6 ± 17.2 (0.5; 60)	0.3154
Mean attack duration [minutes; (min.; max.)]	118.5 ± 216.7 [10; 720]	184.3 ± 249.2 (2; 720)	34.1 ± 48.3 (1; 180)	0.0933
**Accompanying symptoms**				
Headache	3 (30%)	12 (80%)	0 (0%)	**<0.0001**
Speech disturbance	3 (30%)	1 (7%)	0 (0%)	**0.0429**
Limb ataxia	1 (10%)	0 (0%)	0 (0%)	0.2147
Walking difficulties	6 (60%)	6 (40%)	6 (40%)	0.5455
Photo-/Phonophobia	2 (20%)	12 (80%)	0 (0%)	**<0.0001**
Nausea/Vomiting	8 (80%)	9 (60%)	7 (47%)	0.2494
Double vision	2 (20%)	0 (0%)	1 (7%)	0.1752
Oscillopsia	4 (40%)	4 (27%)	2 (13%)	0.3149
Paleness	5 (50%)	2 (13%)	1 (7%)	**0.0212**
Falls	2 (20%)	2 (13%)	2 (13%)	0.8775
**Trigger**				
Sport	6 (60%)	0 (0%)	0 (0%)	**<0.0001**
Stress	3 (30%)	3 (20%)	0 (0%)	0.0951
Position change	0 (0%)	1 (7%)	0 (0%)	0.4254
Loud noise	0 (0%)	1 (7%)	0 (0%)	0.4252
Weather change	0 (0%)	2 (13%)	0 (0%)	0.1730
**Clinical exam**				
Finger nose test	0 (0%)	0 (0%)	0 (0%)	-
Finger chase test	2 (22%)	0 (0%)	0 (0%)	**0.0298**
Dysdiadochokinesis	2 (20%)	1 (7%)	0 (0%)	0.0728
Knee shin test	1 (12%)	0 (0%)	0 (0%)	0.1458
Romberg test	2 (20%)	0 (0%)	0 (0%)	**0.0425**
**Ocular-motor findings**				
Strabismus	7 (70%)	7 (47%)	8 (53%)	0.4349
Spontaneous nystagmus	0 (0%)	0 (0%)	0 (0%)	-
Provocation nystagmus	4 (40%)	2 (13%)	0 (0%)	**0.0226**
Gaze holding nystagmus	5 (50%)	4 (27%)	0 (0%)	**0.0120**
Downbeat nystagmus	4 (40%)	1 (7%)	0 (0%)	**0.0085**
Upbeat nystagmus	2 (20%)	2 (13%)	0 (0%)	0.2273
Rebound nystagmus	3 (30%)	0 (0%)	0 (0%)	**0.0077**
Smooth pursuit saccadic	10 (100%)	7 (47%)	1 (7%)	**<0.0001**
Optokinetic nystagmus path.	6 (60%)	0 (0%)	0 (0%)	**0.0017**
Fixation suppression path.	5 (50%)	1 (7%)	1 (7%)	**0.0076**
Ocular counter-roll	1 (11%)	1 (7%)	0 (0%)	0.4616
**Instrument-based findings**				
Video HIT path.	0 (0%)	0 (0%)	0 (0%)	-
Caloric irrigation path.	0 (0%)	0 (0%)	0 (0%)	-
AEP path.	0 (0%)	0 (0%)	0 (0%)	-
VEMPS path.	0 (0%)	0 (0%)	0 (0%)	-
Audio path.	0 (0%)	0 (0%)	0 (0%)	-
Posturography functional	5 (56%)	1 (14%)	7 (64%)	0.1070

Delayed motoric/cognitive development was considered when children did not achieve the gross motor developmental or cognitive (including language development and intellectual growth) milestones respectively. Statistically significant p-values (<0.05) are displayed in bold.

## Discussion

### EA syndromes

Episodic ataxia syndromes are rare genetic disorders, but as demonstrated here, in a specialized vertigo/dizziness clinic they might be present in about 5% of patients under the age of 18 years (see Figure 1). The most common EA syndrome was EA type 2 followed by EA type 1. Of the seven patients with EA type 2 included in the study, five had mutations that have not been described before (patients 5–9). Additionally, the mutation of the EA type 1 patient (patient 1) also constitutes a novel description (details presented in Table 1). According to criteria suggested by Jen 2008 (8) these new mutations were considered disease-causing, as all newly described mutations were heterozygous and caused a premature stop or altered amino acid residues. The high number of spontaneous mutations in the present study supports the suggestion to consider EA also in children and adolescents with a negative family history.

The patient included here with a mutation in the FGF14 gene is to our knowledge the thirteenth case described with the clinical syndrome of episodic ataxia (7, 15, 16). FGF14 mutations are considered a rare cause of spinocerebellar ataxia type 27 (SCA 27) and show a broad phenotypic spectrum (17–20). The case presented here showed recurrent attacks of vertigo and dizziness lasting between a few minutes and 2 h, accompanied by severe walking difficulties partly leading to falls. Furthermore, the patient had a developmental delay (mental and motor) and psychiatric comorbidity. Further characteristics are presented in Tables 1–3. Overall, this case extends the phenotypic spectrum of EA related to a mutation in the FGF14 gene and further supports the previous suggestion to characterize such patients as EA type 9 (7).

A high genotype-phenotype variability has been shown especially in patients with EA type 2 and EA type 1 (6, 17, 21, 22). Furthermore, mutations in the most common genes associated with EA, namely in the CACNA1A gene (associated with EA type 2) and KCNA1 (associated with EA type 1) might lead to different clinical entities without episodes of vertigo/ataxia, such as epilepsy, paroxysmal dyskinesia, or hemiplegic migraine (4, 6, 21, 23). Also, in a considerable number of patients with a clinical syndrome of EA, no disease-causing mutation might be found (4, 24–26).

### EA phenotype, instrument-based findings, and treatment

Since a broad clinical spectrum has been described in patients with different types of EA, a precise clinical evaluation of patients suspected of suffering from EA is of the utmost importance in order to reach a correct diagnosis and initiate appropriate therapeutic measures including medical treatment option with acetazolamide or 4-aminopyridin.

In the present study, all the EA children/adolescents included suffered from vertigo or dizziness attacks lasting between 5 min and 12 h, in the mean 2 ± 3.6 h The lowest attack frequency was one attack per month, the highest was daily attacks. All children/adolescents had accompanying symptoms, such as paleness, speech disturbances, and walking difficulties. Walking difficulties were often described as the “inability to walk straight” or children/adolescents “lying down and not being able to stand up”. Many children/adolescents reported trigger factors, most frequently physical exercise (“sports”), but two children did not report any triggers. Physical exercise leading to an attack most commonly was described while participating in school sports, but also in lighter activities such as climbing a few stairs. These findings seem to constitute the core symptoms of EA children/adolescents. This is also in line with findings from previous studies and case descriptions of patients with EA (4, 6, 7, 22, 26–35).

In the interictal interval only three children/adolescents had clinical signs of ataxia, two with EA type 2 and the above-described adolescent with EA type 9. As expected, in the latter, ataxia was much more pronounced (see Table 1), since patients with an FGF14 mutation show an overlap between episodic and chronic progressive ataxia (7). No pathological findings were found in any child/adolescent in the instrument-based findings such as vHIT, caloric irrigation, AEP's, VEMPs, or the audiogram (see Table 3).

To our knowledge, this is the first prospective study to evaluate the ocular-motor function of children/adolescents with EA in detail. One previous study retrospectively reported on neuro-ophthalmological findings in chronic ataxia, which included 7 patients with EA and described the presence of nystagmus in these patients, however without further differentiation (36). A second study retrospectively analyzed eye movement disorders in children with CACNA1A mutations, most frequently describing paroxysmal tonic upgaze, saccade dysmetria, and strabismus (37). Strabismus was also present in most children/adolescents (7/10, see Table 3) of our cohort. The prevalence of strabismus among children/adolescents varies between different countries in the world between <1% and 8.6% (38), thus suggesting a much higher prevalence among children/adolescents with EA. Paroxysmal tonic upgaze and saccade dysmetria were not present in our cohort. The most common finding in all patients in the present study was a pathological smooth pursuit, either horizontally, vertically, or in both directions. Furthermore, most children/adolescents showed a nystagmus in the interictal interval, either a vertical spontaneous nystagmus (4/10 down-beat, 2/10 up-beat) and/or a gaze-holding nystagmus (5/10, see Table 3). The optokinetic nystagmus was disrupted in 6/10 children/adolescents and a pathological fixation suppression was found in half of the children/adolescents. These findings are in line with a study describing ocular-motor findings in nearly only adults with EA type 2 (39, 40), so it can be assumed that ocular-motor disturbances are also distinct clinical findings in children and adolescents with EA.

Treatment with acetazolamide or 4-aminopyridine improved attack frequency and duration in some, but not all patients, which is in line with previous findings (8, 24, 28). Both treatments are not approved for use in patients with EA, so it is an individual treatment that children/adolescents and parents must be made aware of. Additionally behavioral recommendations (e.g., reducing physical activity and stress) should not be underestimated, since they might reduce EA attacks.

### Distinction of EA among dizzy patients

The most common differential diagnoses of EA are RVC and more importantly VM. These diseases can show similar vertigo/dizziness attacks and accompanying symptoms such as headaches (see Table 4), so that a distinct differentiation in order to reach the correct diagnosis and subsequently initiate the correct treatment is merited.

Attack frequency was similar in all three patient groups, while attack duration was shorter in RVC with a maximum of 3 h compared to a maximum of 12 h in EA and VM patients. The most common accompanying symptoms of VM attacks were headache, photo-/phonophobia, and nausea/vomiting, which was expected in accordance with the diagnostic criteria (13). EA patients in our cohort showed typical clinical attack features (i.e. sport or stress induced ataxia or vertigo, accompanied by walking-difficulties, oscillopsia, and paleness) as previously described (9, 28). The two included EA type 1 patients had no evidence of myokymia which is often reported in EA type 1 (41, 42). As EA type 9 overlaps with SCA 27, this patient had a high SARA score, while the SARA score was slightly increased in only two EA type 2 patients and none of the EA type 1 patients. Furthermore, RVC patients more frequently showed a functional sway pattern in posturography, which may indicate a higher risk of secondary psychosomatic development, similarly to VM and migraine-related disorders (43, 44).

Ocular-motor findings in children with VM have to our knowledge not been reported previously. However, the most common finding of a slight saccadic smooth pursuit in the present VM cohort is similar to findings in adult VM populations (45, 46). In contrast, ocular-motor deficits were rarely noted in RVC (see Table 4). All EA patients showed considerable ocular-motor deficits compared to only slight deficits in VM. Especially EA type 1 is typically reported to not show any interictal nystagmus (41, 42); however, ocular-motor deficits have to our knowledge not been examined in detail before. In our study we find a clearly saccadic smooth pursuit in both EA type 1 patients, as well as a pathological fixation suppression in one of them (see Table 3). The present findings suggest, that distinct interictal ocular-motor findings are present in the most frequent EA types, nevertheless the number of EA type 1 patients in the present study is low and further evaluation of a larger EA type 1 cohort is needed.

In summary, the findings of the present study suggest that EA patients more often report speech disturbances and paleness as accompanying symptoms during the attacks, while VM patients more often report headaches and photo-/phonophobia. Regarding trigger factors, only patients with EA reported physical activity (sport) as an attack trigger. Pathological findings in the clinical evaluation were almost exclusively found in EA patients. The most striking differences between these groups though are the ocular-motor findings, especially various types of spontaneous nystagmus and/or a pathological smooth pursuit in patients with EA. Nevertheless, ocular-motor disturbances, such as gaze-holding nystagmus and a pathological smooth pursuit might also be found in some patients with VM and only rarely in RVC, but overall to a much lesser extent and scarcely with more than one ocular-motor abnormality.

### Proposed diagnostic criteria for EA in children/adolescents

**Figure 2:**
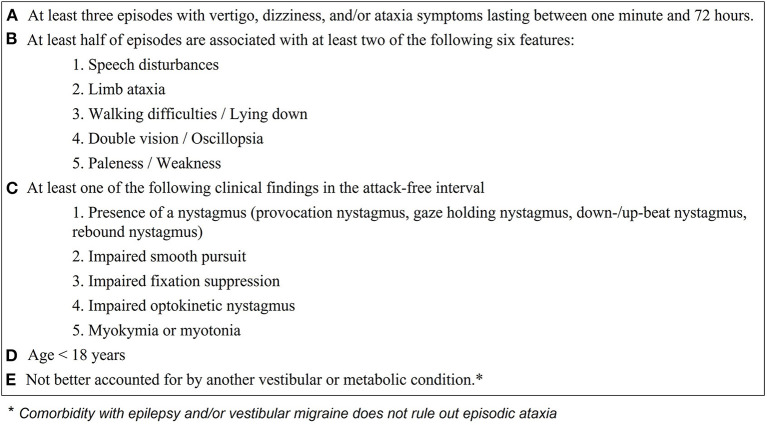
Proposed clinical and ocular-motor criteria for diagnosing children and adolescents with episodic ataxia. With the fulfillment of all criteria **(A–E)** the diagnosis of “probable episodic ataxia” can be made and genetic testing should be initiated. A positive genetic finding confirms the diagnosis of episodic ataxia.

Besides the recommendations by Jen 2004 (4), diagnostic criteria for EA have not been defined. Defining criteria for EA syndromes is especially important, since genetic findings in patients with EA might be negative or inconclusive (4, 24–26). In Figure 2, we propose diagnostic criteria for identifying children/adolescents with “probable EA” according to the findings of the present study, considering a broad literature review (4–12, 15, 16, 22, 24–30, 32–35, 39–42, 47) and our expert opinions. The diagnosis of a “definite EA” should additionally include a pathological genetic finding with a “disease-causing mutation” as characterized by Jen 2008 (8).

The diagnostic criteria include data from medical history such as details on the duration of vertigo attacks and accompanying symptoms (see Figure 2 points A. and B.) as well as ocular-motor findings (see Figure 2, point C). Because ocular-motor findings in EA in some cases might be subtle and only distinguishable by experienced physicians (mostly early in the disease course), we suggest performing a standardized orthoptic examination conducted by a trained orthoptician to evaluate the ocular-motor system. Except gait analysis, which may show an atactic gait in the attack free interval of EA patients, all instrument-based examinations (VEMP's, AEP's, audiometry, posturography, MRI) were unremarkable in EA children, but are necessary to rule out other vestibular conditions (see Figure 2, point E.). We therefore suggest a basic vestibulo-cochlear work-up in all children suspected to suffer from EA comprising of a caloric irrigation, one examination of the auditory function (either AEP's or audiometry), and an MRI. Latter is suggested, since anatomical variants of the vestibular/cochlear organ and central pathologies are not uncommon in childhood (see Figure 1).

When applying the proposed criteria to the present findings, all patients could be identified. Furthermore, the diagnostic criteria were applied on patient descriptions from previous studies of EA including types 1–9 (see Supplementary Table 1). Only studies including a clinical characterization of the attacks as well as interictal findings, at least regarding the presence of nystagmus and/or myokymia/myotonia, were used for analysis. Sensitivity was calculated as the number of correctly identified EA patients (173; including the children of the present study) divided by the number of all EA patients included (223) and reached 78% (Supplementary Table 1). The most frequent reason to not fulfill the proposed diagnostic criteria was a missing interictal clinical finding (see Figure 2, point C.). This might be due to the lack of reported broad ocular-motor findings such as smooth pursuit, optokinetic nystagmus, and fixation suppression, which were only reported in one study (33). Therefore, and according to the present findings, where all EA patients showed considerable ocular-motor deficits, the above reported sensitivity might be underestimated.

Specificity could only be calculated according to the control group defined in the present study (including children with RVC and VM) and reached 90% (3 children with VM fulfilled the here suggested diagnostic criteria). These three children suffered from headache attacks, which might segregate EA from VM. However, we decided not to use “headache attacks” as an exclusion criterium for the suggested diagnostic criteria, since headaches (irrespective of the fulfillment of the diagnostic criteria of VM or other ICHD diagnoses) are commonly present in EA children/adolescents (4, 22, 28). Overall, this suggests a good sensitivity and specificity for the suggested criteria for children with EA; nevertheless the applicability and accuracy must be further evaluated in larger cohorts of precisely characterized children with vertigo, dizziness, or ataxia attacks.

## Conclusion

EA is a rare genetic disorder, but might be present in a considerable amount of children and adolescents presenting with episodic vertigo and/or dizziness. Six new genetic mutations are presented, five for EA type 2 and one for EA type 1, so that a broad genetic testing (e.g., next generation sequencing) in suspected EA patients is recommended. Clinical and instrument-based findings that help to differentiate EA from VM and RVC include attacks triggered by physical activity and accompanied by speech disturbances and paleness. Furthermore, a pathological smooth pursuit and the presence of a nystagmus in the interictal interval seem to be indicative for EA, especially type 1, 2, and 9. In accordance with the findings of the present study, we propose diagnostic criteria for EA, which should be further evaluated as to their sensitivity and specificity for detecting EA in future research.

## Data availability statement

The raw data supporting the conclusions of this article will be made available by the authors, without undue reservation.

## Ethics statement

The studies involving human participants were reviewed and approved by Ethics Committee of the Medical Faculty of the LMU (414-15). Written informed consent to participate in this study was provided by the participants' legal guardian/next of kin.

## Author contributions

FF: patient recruitment, data acquisition, statistical analysis, interpretation of data, and drafting the manuscript. LS and KD: patient recruitment and data acquisition. RS: statistical analysis and interpretation of data. DH: patient recruitment, data acquisition, interpretation of data, and drafting the manuscript. All authors approved the final version of the manuscript.

## Conflict of interest

The authors declare that the research was conducted in the absence of any commercial or financial relationships that could be construed as a potential conflict of interest.

## Publisher's note

All claims expressed in this article are solely those of the authors and do not necessarily represent those of their affiliated organizations, or those of the publisher, the editors and the reviewers. Any product that may be evaluated in this article, or claim that may be made by its manufacturer, is not guaranteed or endorsed by the publisher.
