# Supplementary material for: Episodic ataxias in children and adolescents: Clinical findings and suggested diagnostic criteria
Source: Front Neurol. 2022 Oct 24;13:1016856. doi: 10.3389/fneur.2022.1016856 (PMC9638128; doi:10.3389/fneur.2022.1016856)
Supplement: Supplementary file 1 [file Table_1.pdf]

Supplemental Table 1: Included references and corresponding patient characteristics for sensitivity analysis of the proposed diagnostic criteria for EA. Overall sensitivity was 78%. The lowest sensitivity was reached for rare EA types, such as 7 and 8, mostly due to the absence of distinct interictal clinical features, although precise ocular-motor testing was rarely included.

Ref. = reference number; min = minutes; hrs = hours

			**Criterion A.**	**Criterion B.**					**Criterion C.**			
**Ref.**	EA type	Number of patients	attack duration	speech disturbance	(limb) ataxia	walking difficulties/ imbalance	double vision/ oscillopsia	paleness/ weakness	nystagmus	myokymia	fulfilling new diagnostic criteria	sensitivity
**47**	1	28	(min – hrs)		28	28			-	28	28	1
**41**	1	33	min - hrs	33	33	33			-	17	17	0,52
**4**	2	64	10 min - days		62	(62)		31	60		60	0,94
**22**	2	8	min - days	5	6	6	2		5		6	0,75
**27**	2	2	4 – 6 hrs	2	1		1		2		2	1
**39**	2	28	(hrs)	17	22	6	5		22		22	0,79
**40**	2	3	min - hrs	3	3	3			3		3	1
**25**	2	2	2-3 days	2	2	2			2		2	1
**32**	5	1	hrs		1	1			1		1	1
**33**	6	1	30 min – 3 hrs	1	1				(1 ocular-motor deficit)		1	1
**34**	6	3	hrs	2	2		1		1		1	0,33
**29**	7	1	hrs - 3 days	1	1	1					0	0
**35**	8	10	10 min - 10 hrs	10		10		10	2	1	3	0,3
**7**	9	4	2-7 days		4				2		2	0,5
**15**	9	2	20 min	2	1	2	2		2		2	1
**16**	9	1	min - hrs	1	1		1		1		1	1
**30**	3, 4	22	10 min – 6 hrs		26	7	14			12	12	0,55
**present study**	1,2, 9	10	10 min – 12 hrs	3	1	6	2	5	8 + (2 ocular-motor deficits)	0	10	1
**Total**		**223**									**173**	**0,78**
